# Analysis of Managing Safety in Small Enterprises: Dual-Effects of Employee Prosocial Safety Behavior and Government Inspection

**DOI:** 10.1155/2018/6482507

**Published:** 2018-03-08

**Authors:** Qiwei Wang, Qiang Mei, Suxia Liu, Jingjing Zhang

**Affiliations:** School of Management, Jiangsu University, 301 Xuefu Road, Zhenjiang, Jiangsu Province 212013, China

## Abstract

This paper aims to promote a national and international occupational health and safety (OHS) intervention for small and medium enterprises (SMEs) within internal and external resources. Based on the characteristics of small SME management, the work environment and occupational health may be positively affected by the dual-effects of employees and government. Evolutionary game theory is utilized to identify relevant interactions among the government, small enterprises, and employees. Furthermore, dynamic simulations of the evolutionary game model are used to explore stability strategies and to identify modes of equilibrium.

## 1. Introduction

Occupational health has become a critical issue threatening SMEs in China and across the globe. Accidents, injuries, and associated property damage may have severe consequences for workers and their companies. This reality creates serious concern over occupational health as well as safety issues threatening both society and government. There is urgent demand for effective intervention to minimize accident and injury ratio, especially for SEMs. As of October, 2015, the number of small enterprises in China reached 6.666 million. The European Union reported that micro and small enterprises account for 98.7% of the total of enterprises and employ 50.2% of employees, while large enterprises only account for 1.3% of all enterprises [1]. Small enterprises make significant contributions to Chinese and global economic development. The enormous proportion of small enterprises also represents a large burden upon the government in terms of safety regulations and OHS policies.

Griffin and Neal [2] first defined the concepts of safety behavior, safety compliance, and safety participation. Since then, there has been a wealth of researches on employee safety behavior and its effects on occupational health conditions. The effects of employee safety behavior are well-evidenced by its relationship to injuries and accidents in workplace [3–5]; employees also may perform initiative behavior which enhances the safety performance of their workplace [6, 7]. Employee safety behavior is not only negatively related to accidents and injuries but also may assist the OHS management system in running smoothly [8–10].

Employee prosocial behaviors, by definition, are affiliative in nature and impose positive effects on enterprises. Prosocial behaviors often show the initiative by helping colleagues and taking measures to safeguard their own and welfare. Employees' safety whistleblowing behavior with regard to safety violations is similar to prosocial behavior in its altruistic yet self-interested characteristics [11]. Safety whistleblowing behavior is defined as beneficial for both employees and the enterprise, as it can create personal profit while preventing the illegal production activities [12].

The workplace environments of most small enterprises are riskier on average than those of larger enterprises, and the implementation of safety regulations and laws may be less effective by comparison. Furthermore, because of insufficient recourses and fund, safety management with only a short-term impact in controlling accidents and injuries, this impact also tends to be delayed. Employees who prioritize occupational safety are more likely to blow the whistle on unsafe or illegal production activities, which safeguards not only their own health, but also the development of small enterprise. The government ultimately holds responsibility for regulating the safety of small enterprises as well. Based on current safety policies, the Chinese government empowers employees to whistle blow on enterprises' illegal production activities and encourages them to report related safety information by remitting awards for doing so [13]. Low-cost and universally standard safety management practices for small enterprises have significant potential value. From the perceptive of currently limited safety management capability and insufficient safety investment, this paper combines the effect of employee prosocial safety behavior and government regulations, for example, safety whistleblowing and inspection-practices, to propose an effective safety management practices tailored to small enterprises worldwide.

The safety management characteristics of SMEs are shown as follows. First, SMEs have higher accident and injury rates than large and medium enterprises, not only because SMEs are numerous, but also because safety management is insufficient and noneffective. Second, most SMEs are unable to execute safety policies and laws because of limited resources and lack of safety awareness. Third, there is no expert to fulfill and improve safety management for SMEs, because owner-managers are always safety manager. Considering the safety management characteristics of SMEs, we aim to propose an evolutionary game model to solve safety issues, such as improving safety management in a low-cost method, combing the dual effect of employee prosocial safety behavior and government safety inspection to force SMEs to produce safely, and reducing accident and injury rates for a long period.

The remainder of this paper is structured as follows.  Section 2 provides a literature review on employee prosocial behavior and the characteristics of small enterprises.  Section 3 discusses the research context and methodology utilized in this study.  Section 4 describes the results of evolutionary game simulation by system dynamics.  Section 5 offers a penalty dynamic strategy and optimized penalty-incentive strategy for control over a system in a stable state.  Section 6 provides a brief summary and conclusion.

## 2. Literature Review

Miceli and Near [14] define “whistleblowing” as a behavior performed by an employee or administrative staff, a member who makes certain information public, including personally executed activities or organizational-level activities which may be inherently harmful or in violation of human rights regulations. Whistleblowing is a prosocial behavior that is altruistic in nature. The whistleblowers are tasked with consideration of the consequences to both the enterprise and themselves; whistleblowing may alter the current situation of enterprise safety management or result in a psychological burden on employees. Walters [15] proposed that whistleblowers consider the after-effects of this behavior throughout the decision-making process. The results of whistleblowing may have a negative impact, such as the damage to the reputation of enterprise or punishment exerted upon the whistleblower's colleagues. Whistleblowing behavior may prevent the enterprise managers from making potentially harmful decisions and thus may reduce injuries, accidents, and near-miss incident rates in the workplace [16]. The whistleblower must take stock of the effects of this behavior in terms of economic and social cost at each stage of the process. Employees may be implicitly or explicitly encouraged to keep silent, leaving them no choice but to tolerate an unsafe work environment which puts them at a high risk of injury [17].

Whistleblowing may be “external” or “internal.” External whistleblowing is defined by an employee revealing risky behavior on the part of their company to safety organizations or the government; this includes “anonymous whistleblowing” and “real-name whistleblowing.” By contrast, internal whistleblowing is defined as an employee reporting unsafe behavior or risky conditions to higher management within the enterprise [18]. Internal whistleblowing is less effective in terms of preventing unsafe activities and may even make the workplace even less safe. When enterprise treats unsafe production activities as a common situation, internal whistleblowing is likely to be ignored. Internal whistleblowing also may result in retaliation against the whistleblower. Employees are more likely, to this effect, to report safety violations in their company to outside organizations or government departments. [19].

External safety whistleblowing relates directly not only to employees' occupational health but also to hazardous working conditions and overall limitations on small enterprises. Generally, micro and small enterprises are lacking in social or political support, have limited resources, and are at higher risk of bankruptcy than large enterprises. SMEs owner-managers are also safety managers, which may render the OHS management system ineffective and unprofitable. Safety management systems also tend to suffer from lack of effective intervention, or expert safety guidance; safety training and education are mainly transmitted to employees through informal word-of-mouth [20–22]. Owner-managers are critically tasked with securing sufficient profits to keep the enterprises afloat, so safety may represent an extra burden. Meanwhile, the line of safety communication between owner-managers and employees is shorter in small enterprises. Researches have continually shown that small and medium enterprises have more risky working environments and more work-related injuries and illnesses than large enterprises [23, 24]. The relatively informal structure of small enterprise management also makes external whistleblowing more likely than internal [25, 26]. The OHS of small enterprises is thus impacted under the dual-effects of employees and the government. The management characteristics of micro, small, and medium enterprises are summarized in Table 1.

SMEs have a low safety management due to their economic scale, relatively isolated nature, and the fact that they tend to be geographically dispersed. Employees in small enterprises are domestic, often seasonally employed, and may be relatively unqualified. Safety training and education have limited effect on improving the level of safety at these enterprises, and job security is typically lower than offered by larger enterprises.

Traditional game theory aims to analyze the conflict and cooperation between two players to inform mathematical strategic decision. However, the players must be completely rational and share complete information for the theory to hold; in practice, of course, complete rationality and complete information are rare. Moreover, traditional game theory is not reflective of the dynamic game playing process. Evolutionary game theory, however, combines game theory analysis with a dynamic evolutionary process to seek a dynamic equilibrium, rather than static or comparative static equilibrium. An evolutionary game theory model is normally established based on selection and mutation. Selection identifies higher payoff strategies, which are likely to be adopted by more players, mutation reflects the fact that players choose random strategies to stand out from the group.

We reflect the characteristics of safety management in SMEs by hypotheses of the model and setting of game players. First, we hypothesized that owner-managers had no special relationship with the government. They cannot avoid safety inspection or penalty by bribing government officials. Large and medium enterprises may have loose safety policies or nonstrict safety inspection because of power rent seeking. The model hypotheses reflected the fundamental characteristics of safety management of SMEs. Second, because safety management is insufficient and noneffective, employee prosocial safety behavior and government inspection will show significant impact to prevent unsafe production activities. Therefore, we set the players' strategy according to the characteristic of safety management. Third, large and medium enterprises have safety expert to manage safety; safety issues could be solved through internal whistleblowing. Furthermore, safety management could run smoothly in large and medium enterprises even without safety inspection. However, owner-managers in SMEs manage safety by themselves, employees could only report safety issues to the government. Therefore, we defined the strategy of players according to the characteristic of safety management. Fourth, economic survival and development are two challenges for SMEs. However, large and medium enterprises have well-planned safety investment without the limitation of funds and resources. Therefore, we added specific parameters and coefficients to reflect the characteristic of safety management.

Wang et al. [27] built an evolutionary game model of an environment regulation department and two firms based on dynamic system equilibrium. They found that the penalty strategy can effectively restrain environmental pollution and control fluctuations during the evolutionary game process. However, they did not offer optimal strategy in their evolutionary game model. By comparison, in our study, the game players are rationally bounded and share incomplete information, and they continually adjust their strategies after considering the potential payoffs with others and then adjust their strategies. We not only set evolutionary game model of three game players based on the characteristics of safety management in SMEs but also added penalty-incentive strategy to control the fluctuations. We also find optimal strategy between small enterprises, employees, and the government in our study.

## 3. Hypothesis and Method

Evolutionary game theory can reasonably explain the strategic choices of bounded-rationality players. The advantages of evolutionary game theory include well-represented dynamic evolution behavior, dynamic changes in player behavior, and ultimately reasonable results. Players (government, owner-manager, and employee) adjust their respective strategies by considering the costs and payoffs of every decision. Evolutionary game theory is an appropriate method to explain each player's strategy in different stages of the game.

### 3.1. Game Model Hypothesis and Setting

Owner-managers are mandated to conduct inspections when employees whistle blow on unsafe situations in their workplace. Employees may exercise their civic right to expose those unsafe activities or illegal activities to the government. Punishments, such as suspensions, shut downs, or fines may be handed down to compel the SMEs to improve their safety management practices. The stakeholders' strategy for investing in small enterprises can be represented as an evolutionary game. It is assumed that there is no bribery between the government and small enterprise owner-managers, that the players have incomplete information, and that the government is fully capable of law enforcement. When small enterprise safety issues are reported, the enterprises are fully subject to punishment by the government.

The government is responsible for establishing safety policies which effectively regulate small enterprises in conducting their own safety management and OHS practices. Here, we assume that the government adopts two strategies to fulfill this responsibility: real-time inspection and noninspection. When the government chooses real-time inspection, the inspection cost is *g*1. During inspection process, the government awards enterprises with good safety performance and OHS, at an expected value of *g*2; conversely, the government punishes safety violations at an expected value of *g*3. The government will also award employees who whistle blow unsafe production in their enterprises at an expected value *g*4. If government chooses noninspection, punishment is only handed down upon receiving reports at an expected value *g*5.

When small enterprises have unsafe production situations, accidents and injuries are effectually inevitable. When accidents and injuries occur, the government is passively responsible for them, its own reputation may be damaged, and its administrative capability is questioned by the general public. Real-time inspection on the part of the government or whistleblowing on the part of employees can reduce the ratio of unsafe production at an expected value of *g*6. When the government chooses noninspection and employees ignore unsafe production practices in their enterprise, the accident ratio increases at an expected value of *g*7  (*g*7 > *g*6).

Enterprises have two game strategies: safe production or unsafe production. When enterprises choose safe production, investment in safety increases to a value of *n*1. By contrast, when enterprises choose unsafe production, safety investment is reduced. When accidents happen, enterprises suffer direct and indirect loss: direct loss includes compensation paid to injured employees, penalties paid to the government, and property damage, while indirect loss encompasses the resulting harm to the enterprises' reputation and brand influence. The expected value of loss is *n*2.

Employees have two game strategies in response to their enterprise's production situation: whistleblowing or keeping silent. Employees' expected revenue is directly determined by the enterprise's production situation and OHS. If enterprises choose a safe production strategy, their employees obtain job security and *m*1 represents expected revenue. If enterprises choose unsafe production, their employees are likely to whistle blow (*k* = 1), though they may endure retaliation from the owner-managers when the enterprises are punished or shut down by the government, the expected cost is defined as *m*2. Whistleblowing also has a positive effect in deceasing the accident and injury rates, which gain more job security for the employee at an expected cost of *m*3. When employees choose to keep silent, the accident and injury ratio increase at an expected cost of *m*4.

### 3.2. Probabilities of Game Players' Behavior Strategies

This paper assumes *α*  (0 ≤ *α* ≤ 1) as the inspection ratio of the government. The value of *α* reflects the degree of government inspection, such degree shows strict as *α* increases. When *α* = 0 or 1, the government performs noninspection or real-time inspection, respectively.

Enterprises may choose *β*  (0 ≤ *β* ≤ 1) as their strategy during the production process, where *β* is the safe production ratio. When *β* = 0 or 1, there is unsafe production or safe production, respectively.

Employees may choose *γ*  (0 ≤ *γ* ≤ 1) as their behavior strategy, in which *γ* is the employee whistleblowing ratio. When *γ* = 0, employees keep silent when facing the unsafe production conditions. When *γ* = 1, employees expose unsafe production activities to the government in effort to improve their employer's OHS management system.

Based on the above hypothesis and description and list of symbols in the last section, the payoff matrix of the government, small enterprises, and employees is shown in Table 2.

### 3.3. Game Model Solution and Analysis

Replicator dynamics and the evolutionary stable strategy are two core categories of evolutionary game theory. Replicator dynamics explain the bounded rationality of participants. In the game strategy of safe enterprise production, individuals in the government, employees, and enterprises adjust their dynamic behavior based on the dynamic strategy and profits which result for different subjects per evolutionary mechanism. We assume that the fitness ratio of government real-time inspection is $\bar{R_{g,\alpha }}$, and the fitness ratio of noninspection is $\bar{R_{g,1-\alpha }}$.

(1) Rg,α¯=β−g1−g2+1−βγ−g1+g3−g4−g6+1−β1−γ−g1+g3−g6,

(2) Rg,1−α¯=1−βγg5−g6−1−β1−γg7.

The average fitness ratio of government inspection $\bar{R_{g}}$ is as follows.

(3) $$\begin{array}{c} \bar{R_{g}}=\alpha \bar{R_{g,\alpha }}+\left(\;1-\alpha \;\right)\bar{R_{g,1-\alpha }}. \end{array}$$

This paper assumes that the government tends to choose the most beneficial game strategies throughout the game. When the strategy is more beneficial, other game subjects are impacted by the strategy. Here, we assume the inspection ratio of government is *a*; the current inspection ratio imposes different effects compared to pure strategy fitness and average strategy fitness in the subsequent stage. The replicator dynamics equations of government inspection are expressed as follows:

(4) Fα=dαdt=αRg,α¯−Rg¯=α1−α∗β−g1−g2+1−βγ−g1+g3−g4−g5+1−β1−γ−g1+g3−g6+g7.

Plugging (1) and (2) into (4), yields (5) and (6), which represent the replicator dynamics equation of enterprises' safe production and employees' whistleblowing, respectively.

(5) Fβ=dβdt=β1−β∗α−n1+n2+g2+g3−1−αn1+1−αγn2+g5+1−α1−γn2,

(6) Fγ=dγdt=γ1−γ∗α1−β−m2+m3+g4+1−α1−β−m2+m3−βm1+1−βm4.

Based on (4), (5), and (6), the replicator dynamics equations of small enterprises, employees, and the government are shown as follows:

(7) dαdt=α1−αβ−g1−g2+1−βγ−g1+g3−g4−g5+1−β1−γ−g1+g3−g6+g7,dβdt=β1−βα−n1+n2+g2+g3−1−αn1+1−αγn2+g5+1−α1−γn2,dγdt=γ1−γα1−β−m2+m3+g4+1−α1−β−m2+m3−βm1+1−βm4.

The replicator dynamics equation reflects the strategy adjustment speed and ratio between the government, small enterprises, and employees. When the result of the replicated dynamic equation is zero, the system reaches a relatively stable status. Based on Friedman's theory [28], the stability equilibrium points of replicator dynamics equation can be determined by analyzing the equilibrium points of the Jacobian matrix and eigenvalues. Analyzing all the equilibrium points of System (7), unfortunately, not only it comes with a massive calculation workload, but also it does not readily reveal the game subjects' strategies. Computer simulation method better reflects appropriate strategies according to dynamic players models.

## 4. SD-Based Evolutionary Game Simulation

**Figure 1:**
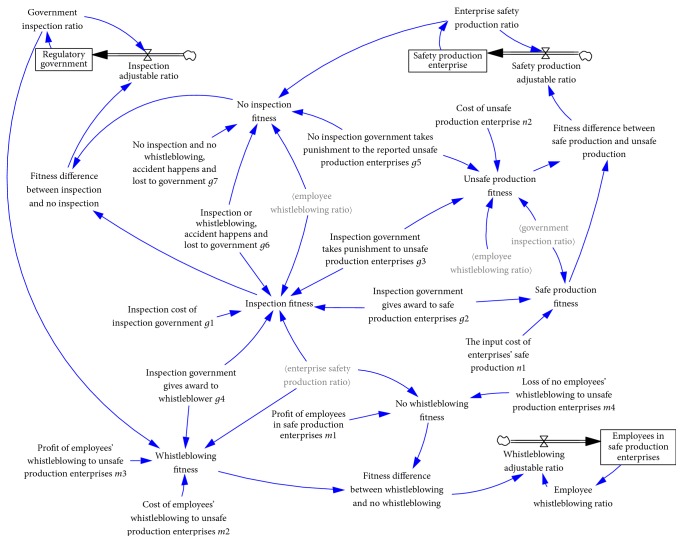
Evolutionary game SD model between government, enterprises, and employees.

The stability of equilibrium points is usually analyzed by system dynamics when researching a tripartite game system [29–31]. Here, we built an SD model of the evolutionary game in Vensim PLE 6.0 software to analyze the game model described above. The game system is made of three submodels: the government inspection sub-SD model, small enterprises safe production sub-SD model, and employees prosocial behavior sub-SD model, as shown in Figure 1. The function relationship between state variables, flow variables, and intermediate variables is based on the above replicator dynamics equation.

Table 3 shows the initial model settings: initial time = 0, final time = 1, time step = 0.03125, and integration type: Euler.

Stability strategy combinations result from taking various parameters in the replicated dynamics equation. The system has eight equilibrium points of pure strategy and two points of mixed strategy as follows:

(8) X1=000,X2=010,X3=011,X4=001,X5=100,X6=101,X7=110,X8=111,X9=0.50.250,X10=00.60.5.

**Figure 2:**
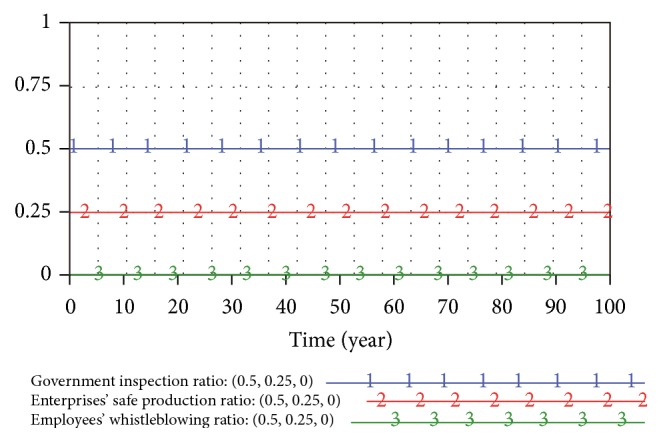
Game results when considering point *X*_9_.

Take the point *X*_9_ = (0.5,0.25,0)^*T*^ as an example, which reveals the stability status of the evolutionary game model by SD simulation. As shown in Figure 2, the tripartite subjects do not change their initial strategy initiatively in the equilibrium point of *X*_9_. Namely, each player maintains their strategy, and the game model is stable.

**Figure 3:**
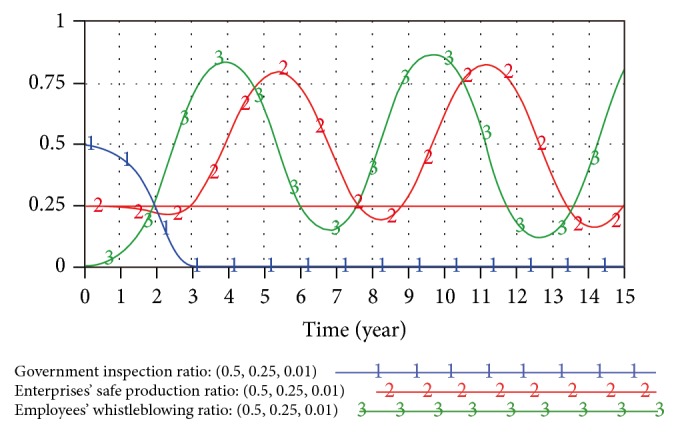
Game results under mutation of *X*_9_.

The equilibrium point is not stable but instead is path dependent. In the initial mixed equilibrium point *X*_9_, when the probability of game subjects mutates slightly (e.g., the mutation rate of employee whistleblowing *γ* = 0.01), the resulting simulation is as shown in Figure 3.

The simulation results show that mixed strategy equilibrium point *X*_9_ is not stable. The government inspection ratio changes with the status of *γ* = 0, while the game probability of enterprises and employees periodically fluctuates (i.e., representing no stable strategy). The mixed strategy equilibrium point *X*_10_ and other pure strategy equilibrium points are likewise unstable. The game model process has repeated fluctuations and altogether unstable development trends according to our SD analysis.

## 5. Stability Analysis and Dynamic Control Scenario

**Figure 4:**
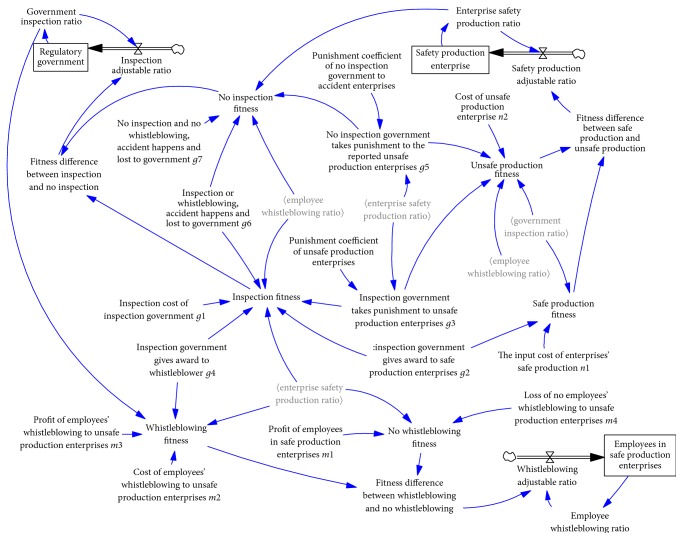
Evolutionary game SD model under the dynamic penalty system.

System fluctuations severely complicate the design of a reasonable trilateral game strategy, so we prioritized them in designing our stable evolutionary game model. Researchers in [27, 32] have attempted to control fluctuations in the game process according to the fact that unsafe enterprise production can be effectively restrained by penalties. We applied the following dynamic penalty control strategy to control fluctuations, with special focus on scenarios in which the government takes a penalty to reduce unsafe production, which are shown as follows:

(9) g31=p11g31−β,

(10) g51=p12g51−β,

where *p*_11_ and *p*_12_ represent the penalty ratio of unsafe production when there is real-time inspection versus noninspection. The values are all given as 1 to simplify the analysis. The SD model of the evolutionary system is shown in Figure 4.

**Figure 5:**
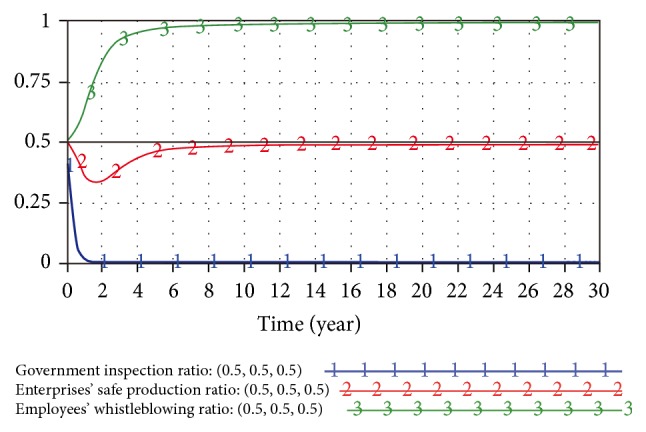
Game model result under initial strategy (0.5,0.5,0.5).

**Figure 6:**
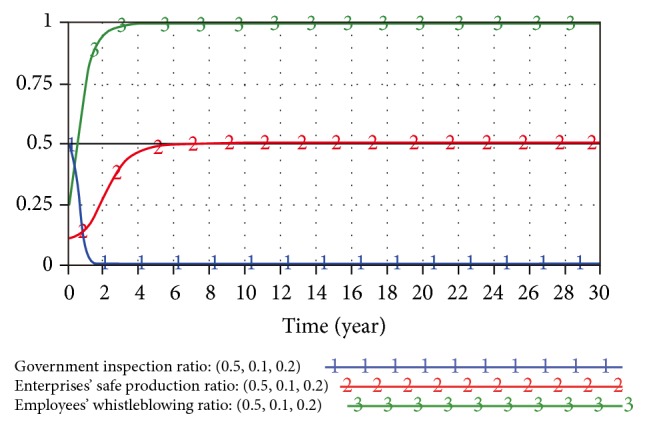
Game model result under initial strategy (0.5,0.1,0.2).

When the initial strategy of the trilateral game model is (0.5,0.5,0.5) and (0.5,0.1,0.2), the SD game results are as shown in Figures 5 and 6.

The evolutionary game model is effectively controlled under the dynamic penalty. The game process tends to convergence (0,0.5,1), which means that government is very likely to select the noninspection. Small enterprises tend to product safely at ratio of 0.5 and employees are also likely to report unsafe production. However, this evolutionary stability strategy is not the optimal strategy; small enterprises still have a high probability of unsafe production.

We next added a penalty-incentive method to control the game model based on the above description [33–35]. In this model, the government chooses a dynamic penalty in response to unsafe production and offers dynamic incentives for employee whistleblowing, and the equations are shown as follows:

(11) g32=p21g31−β+q21n1α,g52=p22g51−β+q22n1α,

(12) g22=p23g2β+q23αn1,g42=p24g4β+q24αm3,

where the coefficient of *p*_21_, *q*_21_, *p*_22_, and *q*_22_ represents the penalty ratio of unsafe production when the government chooses real-time inspection and noninspection, respectively. The coefficient of *p*_23_, *q*_23_, *p*_24_, and *q*_24_ represents the incentive of enterprises for safe production and employee whistleblowing, respectively. Again, all values are given as 1 to simplify the analysis.

**Figure 7:**
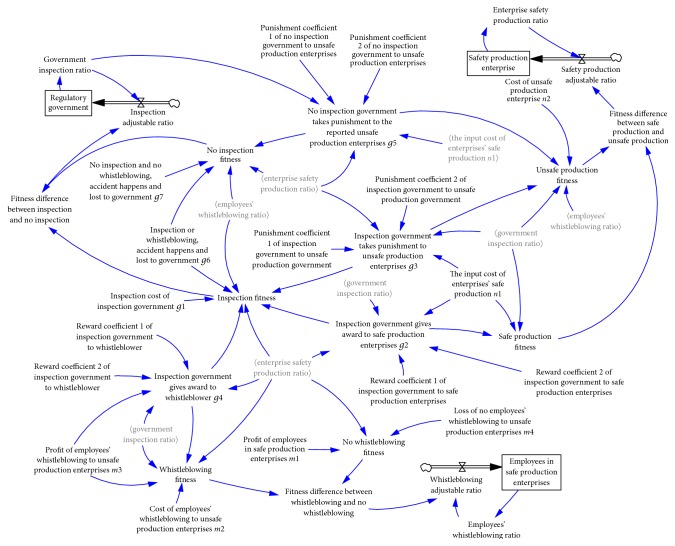
Evolutionary game SD model under dynamic penalty-incentive system.

The SD model of this evolutionary game is shown in Figure 7.

**Figure 8:**
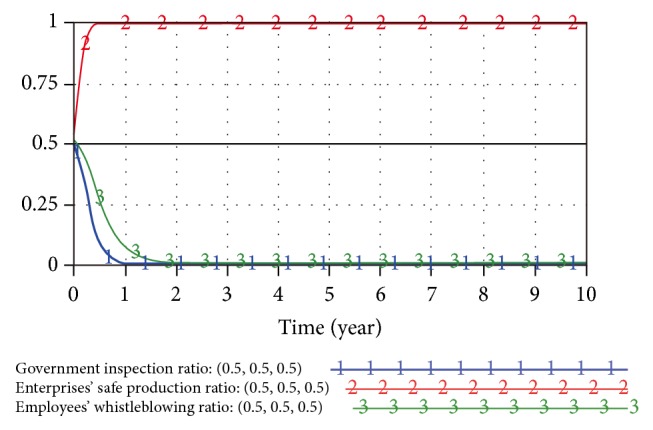
Game result under dynamic penalty-incentive strategy (0.5,0.5,0.5).

**Figure 9:**
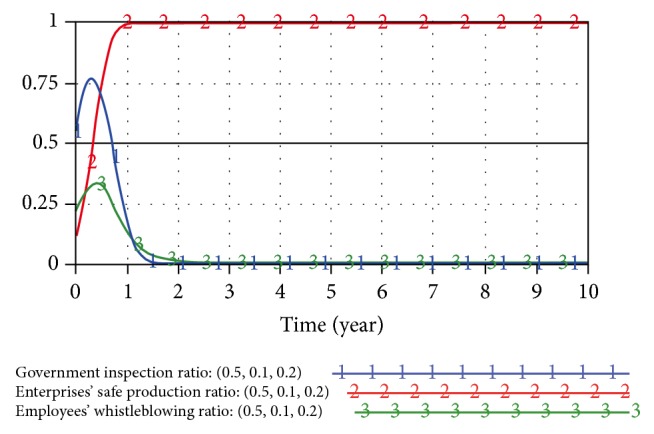
Game result under dynamic penalty-incentive strategy (0.5,0.1,0.2).

When the initial strategy of the trilateral game model is (0.5,0.5,0.5) and (0.5,0.1,0.2), the SD results are as shown in Figures 8 and 9.

Based on the simulation results in Figures 8 and 9, the result of evolutionary game process has a convergence (0,1, 0), at which point the table status is optimal. Namely, the government has an extremely small inspection ratio to small enterprises' production processes; enterprises will follow safety policies, and employees are extremely unlikely to whistle blow.

We next calculated the equilibrium point of the evolutionary game model to validate the above simulation result. Convergence occurs at point (0,1, 0); its stability is proven below.

The Jacobian matrix at the equilibrium point is as follows:

(13) J=∂Fα∂α∂Fα∂β∂Fα∂γ0∂Fβ∂β∂Fβ∂γ00∂Fγ∂γα→0,β=1,γ=0=−6+353α+α23−1−αα26+600−α26−5α−4000−3.

The eigenvalues are as follows:

(14) λ1=−6+353α+α23<0,λ2=−α26−5α−4<0,λ3=−3<0.

Thus, the equilibrium point (*α*, 1,0)  (*α* → 0) represents the stable evolutionary strategy, in accordance with the SD model.

The evolutionary game process and system dynamics described here are indeed effective in analyzing the stability of equilibrium solutions. Our simulation results indicate that the dynamic penalty model can effectively control fluctuations and ensure a stable game. The dynamic penalty-incentive model also offers a stable solution for controlling fluctuations and yields an optimal evolutionary strategy, wherein small enterprises tend to choose safe production, government tend to choose noninspection, and employees tend to choose keeping silent.

## 6. Conclusion and Implications

There is a constant battle between production and safety seemingly inherent to small enterprises. Namely, OHS management system in small enterprises is relatively poor, compared to large enterprises, and may have delayed effect in regulating safety. Small enterprises face various issues threatening development and survival; solutions of safety issues must not worsen these issues and must work alongside factors related to their employees and the government [36].

We used evolutionary game theory which is utilized to explore the multiplayer system at work in the above dynamic game model, comprised of employees, small enterprise, and the government. The calculation burden of analyzing the stability of equilibrium points via the Jacobian matrix is costly and difficult. By contrast, system dynamics can readily yield equilibrium solutions, while effectively controlling stability.

Further, stable evolutionary strategies for the government, small enterprises, and employees are difficult to design and implement during the analysis process. We introduced the dynamic penalty model to control fluctuations and reveal important factors at work in the game. We also added a dynamic penalty-incentive control model and proved that the evolutionary game model, and optimal cases of gameplay, can be fully explained accordingly.

**Figure 10:**
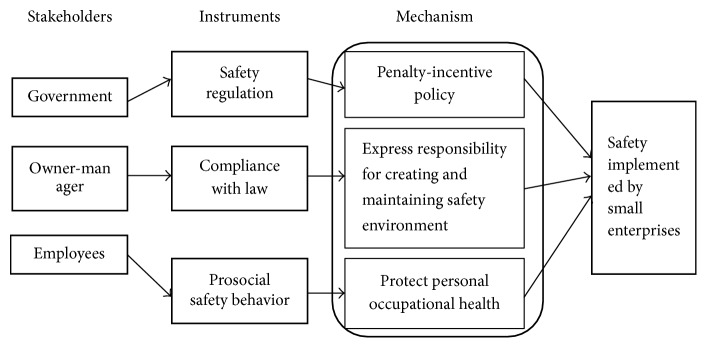
Conceptual model of safety intervention programs for small enterprises.

SMEs are numerous and geographically dispersed, which makes executing successfully any intervention very challenging. There are two main principles governing OHS improvements or reduction of hazardous working conditions: low-cost safety investment and mutual efforts [37–39]. Based on our simulation and system dynamic results, safety intervention programs for SMEs can be illustrated via conceptual model (Figure 10). This model includes three stakeholders tasked with selecting independent instruments: the government has responsibility to inspect the occupational health and working conditions of small enterprises; owner-managers must comply with the given safety regulations; and employees have the right to whistle blow on the dangerous production activities or safety hazards. These three instruments build the mechanisms, which hold small enterprises accountable for safety under penalty-incentive policies and common standards, while employees are accountable for understanding their civil rights with regard to occupational safety [40].

The results of this research can be summarized as three main points.

1. Simulation results suggest that the government does not need to inspect the small enterprise safety situations in real time. Namely, safety regulations should be less restrictive for small enterprises than large enterprises. There are many more small than large enterprises in any given country; non-real-time regulation or tailored inspection strategies work well while imposing relatively little pressure on politicians and decision makers. OHS management systems are not readily accepted by small enterprise owner-managers, so the government would do well to offer financial support or safety technologies (including training, meetings, or seminars) to help small enterprise establish OSH management systems as opposed to simply punishing them for lacking such systems. Issuing rewards to small enterprises with good safety performance and strong OHS management also may better stimulate safety initiatives from within small enterprises.
2. Small enterprises are regulated by two hierarchical levels encompassing penalty-incentive policies and employees' prosocial behavior (e.g., whistleblowing). Therefore, safety performance and working conditions are effectively improved and stably maintained by dual efforts of the government and employees. Owner-managers of small enterprises should prioritize communication with employees and encourage them to voice any concerns regarding safety in the workplace.
3. When the work environment is safe and occupational injuries are very rare, employees will not whistle blow. Employees should still mind their civil rights instead of keeping silent in order to force owner-manager to follow the safety rules. A whistleblowing-free environment also reduces the psychological burden and work-related stress on employees to contribute to an altogether safer and healthier workplace. Employees should voluntarily participate in safety training to gain the safety knowledge they need to quickly and accurately identify unsafe activities.

OHS management systems need investment; though small enterprises owner-managers may believe that such investment does not equal benefit [41]. It is important to note that the characteristics of large enterprises and SMEs inherently differ. Further research is necessary to better tailor OSH management system designs and safety regulations to small enterprises specifically and to better understand how safe production affects political decisions and employee safety behavior.

## Figures and Tables

**Table 1:** Management characteristics of micro, small, and medium enterprises.

Items	Micro enterprises	Small enterprises	Medium enterprises
Safety manager	Owner-manager	Owner and unprofessional manager	Professional manager
Effects of safety management	Ineffective management, owner participates in operations	Effective management	Better and more effective management, full-time management level
Status of development	Very low profit and low growth	Stabilized profit and growth	Strong profit and growth
Safety status	Rife with operation and safety issues	Less operation and safety issues	Small number of operation and safety issues
Safety standard	Nonstandard operation and safety practices	Some formalized operation and safety practices	Formalized operation and safety practices

**Table 2:** Payoff matrix of government, small enterprise, and employees.

Strategic profile	Employee whistleblowing	Keeping silent	Players
Government real-time inspection			
Enterprises' safe production	—	−*g*1 − *g*2	Government
	—	−*n*1 + *g*2	Enterprises
	*m*1	*m*1	Employees
Unsafe enterprises' production	−*g*1 + *g*3 − *g*4 − *g*6	−*g*1 + *g*3 − *g*6	Government
	−*n*2 − *g*3	−*n*2 − *g*3	Enterprises
	−*m*2 + *m*3 + *g*4	−*m*4	Employees
Noninspection			
Safe production	—	0	Government
	—	−*n*1	Enterprises
	*m*1	*m*1	Employees
Unsafe production	*g*5 − *g*6	−*g*7	Government
	−*n*2	−*n*2 − *g*5	Enterprises
	−*m*2 + *m*3	−*m*4	Employees

*Note*. — indicates a lack of no game combination, so it has no payoff.

**Table 3:** Initial SD model parameters.

Parameters	Initial values
*g*1	5
*g*2	1
*g*3	3
*g*4	1
*g*5	4
*g*6	3
*g*7	7
*n*1	6
*n*2	4
*m*1	3
*m*2	2
*m*3	1
*m*4	4

## Symbols

*g*1: Government inspection cost
*g*2: Expected value of award from government to safe enterprise
*g*3: Expected value of punishment from government to unsafe enterprise
*g*4: Expected value of the award of government to employees' whistleblowing
*g*5: Expected value of punishment of government only receiving report from employees
*g*6: Reducing ratio of unsafe production
*g*7: Increasing ratio of unsafe production
*n*1: Increasing safety investment
*n*2: Direct and indirect loss of enterprise
*m*1: Employees' expected revenue
*m*2: Expected cost of damage of employee whistleblowing
*m*3: Expected cost after whistleblowing
*m*4: Expected cost of keeping silent
*α*: Inspection ratio of government
*β*: Enterprise strategy of production
*γ*: Employees strategy of safety behavior
$\bar{R_{g,\alpha }}$: Fitness ratio of government real-time inspection
$\bar{R_{g,1-\alpha }}$: Fitness ratio of government noninspection.
